# Classical and Novel Lipid-Lowering Therapies for Diabetic Patients with Established Coronary Artery Disease or High Risk of Coronary Artery Disease—A Narrative Clinical Review

**DOI:** 10.3390/ph17050568

**Published:** 2024-04-29

**Authors:** Nikolaos Velidakis, Panagiotis Stachteas, Evangelia Gkougkoudi, Christodoulos Papadopoulos, Nikolaos P. E. Kadoglou

**Affiliations:** 1 Medical School, University of Cyprus, 2029 Nicosia, Cyprus; velidakis.nik@gmail.com (N.V.); gkougkoudi.evangelia@ucy.ac.cy (E.G.); 2 Third Department of Cardiology, Aristotle University of Thessaloniki, General Hospital “Hippokration”, 541 24 Thessaloniki, Greece; staxteasp@gmail.com (P.S.); chrpapado@gmail.com (C.P.)

**Keywords:** diabetes, statins, ezetimibe, PCSK9 inhibitors, fibrates, lipid lowering

## Abstract

Diabetic atherosclerosis is a complex process that is characterized by diffuse and unstable lesions increasing 2–4-fold the risk of adverse cardiovascular (CV) events. Diabetic dyslipidemia has a predominant role in coronary artery disease (CAD) and has been the target of classical and emerging pharmaceutical agents with established or promising CV benefits. The aim of the present narrative review was to summarize the effects of classical and novel lipid-lowering pharmaceutical agents on lipid profile and CV outcomes in diabetic patients with established CAD or high risk of CAD. Statins remain the first-line treatment for all diabetic patients since they considerably ameliorate lipid parameters and non-lipid CV risk factors, leading to reduced CV morbidity and mortality. Complementary to statins, ezetimibe exerts lipid-lowering properties with modest but significant reductions in major adverse cardiovascular events (MACEs) and CV mortality. PCSK9 inhibitors considerably reduce LDL-C levels and lower MACEs in diabetic patients. On the other hand, fibrates may confer a very modest decline in MACE incidence, while the CV impact of omega-3 fatty acids is promising but remains questionable. Bempedoic acid and inclisiran have a potential therapeutic role in the management of diabetic dyslipidemia, but this is still not adequately documented. Given the heightened CV risk among individuals with diabetes, more decisive results would be of great importance in the utility of all these drugs.

## 1. Introduction

Diabetes mellitus (DM) is one of the most common chronic metabolic diseases, and accounts for over 10.5% of the adult population [1]. Its incidence is exponentially increasing, but most importantly ranks among the top causes of morbidity and mortality worldwide according to the World Health Organization (WHO) [2], predominantly due to the premature development of atherosclerotic cardiovascular disease (ASCVD), including coronary artery disease (CAD) [3]. It is well established that DM and ASCVD commonly co-occur, and diabetic patients show a 2-fold and 4-fold higher risk for CAD and stroke, respectively, compared to non-diabetic subjects [2]. It is not unusual the diagnosis of type 2 DM (T2DM) to come shortly after an ASCVD manifestation and, vice versa, in patients with established T2DM, overt ASCVD or subclinical atherosclerotic lesions often co-exist (e.g., past silent myocardial infarction—MI) [4]. The development of ASCVD in diabetic population depends on the duration of the disease [5] and the presence of additional traditional or newer cardiovascular (CV) risk factors [6], like dyslipidemia [7].

Diabetic dyslipidemia is described as an abnormal lipid metabolism marked by elevated levels of atherogenic lipoproteins and associated with accelerated atherosclerosis development [3]. In this lipid profile, very-low-density lipoproteins (VLDLs), chylomicrons, and small, B100-rich, dense low-density lipoproteins (sdLDLs) are typically quantified in the bloodstream as non-high-density lipoprotein cholesterol (non-HDL-C). Moreover, triglycerides (TG) and triglyceride-rich lipoproteins are significantly elevated [8]. This pattern of lipid disorder is frequently observed in individuals with T2DM, but it is less common in type 1 DM (T1DM) patients, unless their glycemic control is suboptimal [7]. The underlying pathogenetic pathways of diabetic dyslipidemia include not only hyperglycemia, but also insulin resistance, hyperinsulinemia, and abnormal adipokine levels [9]. 

There is an unmet need of reducing early morbidity due to ASCVD in the DM population, a goal that has already been prioritized in the WHO Agenda 2030 for sustainable development (Target 3.4) [10]. However, although statins are the fundamental pillar of diabetic dyslipidemia management with very good results in LDL-C lowering, many high-risk patients under statin monotherapy do not achieve the recommended targets (roughly 80% according to the multinational observational Santorini study [11]). So, combination treatments with other lipid-lowering agents (e.g., ezetimibe) or novel agents (e.g., PCSK9 inhibitor) are strongly warranted to achieve lower CV risk [12]. On the other hand, although the fundamental approach in treating diabetic dyslipidemia has consistently been the effective (and aggressive) control of LDL-C levels [12], other components of this metabolic abnormality like elevated levels of TGs have been independently associated with increased residual CV risk, and thus TGs should be considered as a separate therapy target [13]. There is still an increasing need to develop and apply more effective medications for the primary and secondary prevention of cardiovascular diseases (CVDs) in the DM population.

The aim of this clinical narrative review is to provide a comprehensive analysis of the current evidence on classical and novel lipid-lowering therapies in diabetic patients with CAD or at high risk of CAD. Based on observational and randomized large-scale clinical trials, we summarized the impact of hypolipidemic medications primarily on lipid profile and CV outcomes in patients with T2DM, and secondarily on safety profiles and clinical outcomes.

## 2. Guidelines for Dyslipidemia Therapy in Patients with Type 2 Diabetes

Regardless of underlying causes, the effective control of LDL-C levels has consistently been a fundamental approach in treating dyslipidemia according to current guidelines provided by many prominent scientific organizations such as AHA/ACC, ADA, and ESC [12,14,15]. In patients with DM and mixed lipid disorders, a secondary objective of reducing non-HDL-C levels is also advisable, although there is scarce evidence from clinical trials to support the prognostic benefit of this approach [12].

CV risk categorization determines the LDL-C targets in the management of diabetic dyslipidemia [12], and according to 2023 ESC guidelines for the management of ASCVDs, patients with T2DM should be grouped into the following four CV risk categories [12]:

- Very high CV risk: Individuals with T2DM with established ASCVD or severe damage of target organs, or an estimated 10-year CVD risk above 20% using SCORE2-Diabetes
- High CV risk: Individuals with T2DM without established ASCVD or severe damage of target organs, but an estimated 10-year CVD risk of 10–20% using SCORE2-Diabetes
- Moderate CV risk: Individuals with T2DM without established ASCVD or severe damage of target organs, but an estimated 10-year CVD risk of 5–10% using SCORE2-Diabetes
- Low CV risk: Individuals with T2DM without established ASCVD or severe damage of target organs, but an estimated 10-year CVD risk below 5% using SCORE2-Diabetes

**Figure 1:**
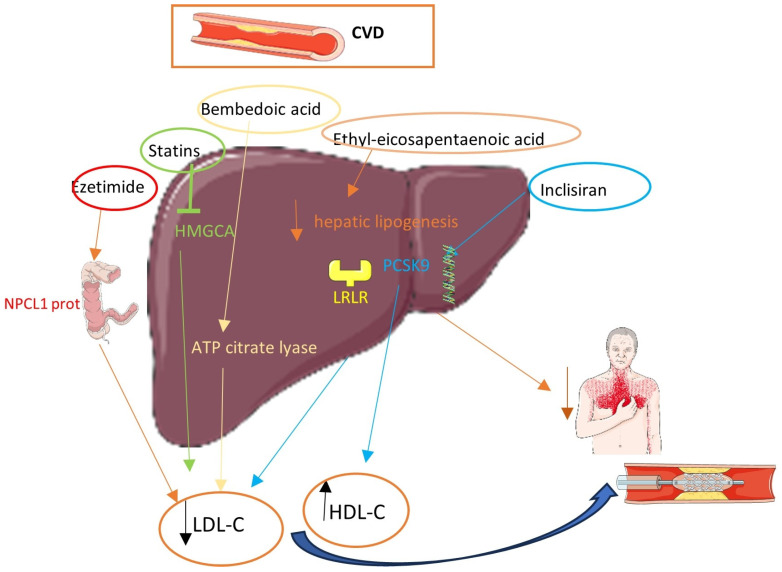
Lipid-lowering therapies and their mechanisms.

Diabetic patients categorized as very high, high, and moderate risk should attempt to achieve LDL-C levels below 55, 70, and 100 mg/dL, respectively (Class I) [12]. Ιn the first two groups, a >50% decrease in LDL-C levels is an alternative target. Therefore, diabetic patients with a history of CAD belong to the very-high-CV-risk group and should be treated aggressively [12]. According to current ESC guidelines, statins are the fundamental pillar of this very-high-risk group and a rapid dosage with up-titration is suggested [12]. If the desired LDL-C level cannot be attained through statin monotherapy, combination treatment with ezetimibe is advisable. In the case of persistently high LDL-C levels despite a maximum-tolerated statin dosage in combination with ezetimibe, or intolerance to statins, the use of a PCSK9 inhibitor is strongly recommended [12] while the implementation of other lipid-lowering therapies is under investigation. Figure 1 depicts the most well-known lipid-lowering medications and their mechanisms.

## 3. Classical Lipid-Lowering Medications

### 3.1. Statins

Until now, statins have been a cornerstone in the management of dyslipidemia in both diabetic and non-diabetic populations [16]. They inhibit 3-hydroxy-3-methyl-glutaryl-coenzyme A (HMG-CoA) reductase, reduce the endogenous production of LDL-C in the liver, and enhance the clearance of atherogenic lipoproteins, leading to a significant decrease in serum total cholesterol (TC) levels and LDL-C levels, and to a lesser extent triglycerides [7]. The different potency between statins, as well as their dose-dependent effects, are extensively studied, with rosuvastatin and atorvastatin exerting the highest lipid-lowering effects in this category [17].

#### 3.1.1. Statins and Cardiovascular Outcomes

Accumulative evidence from randomized controlled trials (RCTs) and observational studies has demonstrated that statins are the most effective medication for mitigating ASCVD burden [18,19]. According to a population-based cohort study among DM patients with established CVD, statin-induced reduction in LDL-C levels was associated with a declined recurrence of CV events and all-cause mortality by 42% (HR: 0.58; 95% CI: 0.42, 0.80), compared to non-statin users [20]. On the other hand, a 4D study showed that diabetic patients with end-stage chronic kidney disease (CKD) treated with atorvastatin 20 mg had a similar incidence of MACEs compared to placebo-treated controls after 4 years of follow-up. Perhaps ASCVD pathogenesis in diabetic patients with CKD involves additional atherogenic mechanisms beyond dyslipidemia [21].

More robust evidence of the cardioprotective impact of statins has been derived from large systematic reviews and meta-analyses. Findings from the Cholesterol Treatment Trialists’ (CTT) Collaboration, from 14 RCTs and more than 18,500 patients with DM, revealed that statin therapy led to a statistically significant 20% reduction in the 5-year incidence of MACEs for each mmol/L decrease in LDL-C levels [22]. A recent meta-analysis assessed the impact of statin therapy on secondary CVD prevention by pooling data from 24 studies of diabetic patients. It demonstrated that statin-treated subjects experienced a significantly lower risk of CVD compared to statin-free individuals (RR: 0.75; 95% CI: 0.65, 0.87; *p* < 0.0001) [23]. Notably, in the same meta-analysis, statin therapy failed to significantly reduce all-cause mortality (RR: 0.85; 95% CI: 0.70, 1.03; *p* = 0.065) [23], which may be related to participants’ backgrounds of CAD or other CVD. A possible explanation for the remarkable CV benefits of statins derives from their pleiotropic effects beyond lipid lowering [24,25], for instance, the favorable modification of the adipose tissue derivatives adipokines, significant amounts of which are found in the blood circulation of diabetic patients with CVD [26].

#### 3.1.2. Aggressive vs. Conventional Statin Therapy

Most importantly, the efficacy of statins seems to be strongly associated with the prescribed dose, presumably by promoting plaque stabilization [27,28]. Patients receiving high-intensity statins (atorvastatin at 40 or 80 mg/dL and rosuvastatin at 20 or 40 mg/dL) achieved a one-third greater reduction in ASCVD risk compared to moderate-intensity statins, making them the preferred choice for individuals deemed to be at high CV risk, like diabetic patients with CAD [29,30,31]. The IDEAL trial recruited more than 8500 patients with established CAD (12% with concomitant DM). Atorvastatin 80 mg/day administration reduced the recurrence of CV endpoints (HR: 0.89; 95% CI: 0.78, 1.01) compared to the less intensive therapy with simvastatin 20 mg/day. However, that association was marginally statistically non-significant [32]. A secondary analysis from the Treating to New Targets (TNT) trial in patients with T2DM and CAD confirmed a clinically significant reduction in MACEs by 25% (HR: 0.75; 95% CI: 0.58, 0.97; *p* = 0.026) in patients subjected to a more intensive statin therapy (atorvastatin 80 mg/day) compared to conventional lipid-lowering treatment (atorvastatin 10 mg/day) [33]. A post hoc analysis of the TNT trial demonstrated that each single component of metabolic syndrome (high BMI, elevated triglyceride levels, low HDL-C levels, hypertension, and increased fasting plasma glucose) relates to an elevated risk of MACEs, but high-intensity statin therapy can only partially mitigate the residual CV risk attributable to these components [34]. A more recent secondary analysis of the TNT trial showed a proportional association between elevated TG levels and CV risk (up to 50%), where aggressive lipid lowering (atorvastatin 80 mg/day vs. 10 mg/dL) could significantly reduce CV risk via TG-mediated mechanisms [13]. This was supported not only by the greater benefits observed in diabetic patients with higher triglyceride levels at baseline, but primarily by the fact that the CV risk reduction was not contingent on decreases in LDL-C levels [13]. Therefore, the beneficial CV impact of statins in diabetic dyslipidemia might be mediated by the amelioration of most lipid parameters—not just LDL-C—and other pro-atherogenic factors (e.g., inflammatory factors).

#### 3.1.3. Adverse Effects of Statins

Statins are generally considered as a safe and well-tolerated hypolipidemic medication [19,35]. Women tend to experience adverse effects more frequently than men, more often referencing symptoms (fatigue, myalgias, and nervous system difficulties) than objective disturbances (significant elevations in creatine kinase, indicative of muscle injury or rhabdomyolysis) [35]. This is often attributed to the nocebo effect [36]. Regarding statin-associated muscle symptoms, moderate-dose statins seem generally better tolerated than high doses of statins. However, accumulative data from observational and randomized trials do not support this assumption [18,37,38]. Some factors predisposing patients to statin-associated myalgias are frailty, drug interactions, excessive alcohol consumption, and vitamin D deficiency [18]. Although it seems rational, a proportional relationship between statin dosage and the occurrence of side effects has not yet been proven.

### 3.2. Ezetimibe

By combining ezetimibe with a statin, an adjunctive reduction in serum LDL-C levels by approximately 20–25% can be achieved [39,40], targeting the inhibition of cholesterol absorption from the gut (ileum) [41] through the suppression of the Niemann-Pick C1-like 1 (NPCL1) protein [42].

#### 3.2.1. Effects of Ezetimibe on Lipid Profile

Hence, ezetimibe is considered a more effective hypolipidemic treatment than up-titrating statin dosage (doubling statin dosage further decreases LDL-C concentrations by 5–7%) [43,44]. In addition to LDL-C lowering, ezetimibe has demonstrated a beneficial profile in diabetic dyslipidemia [45] by reducing triglycerides and sdLDL particles and increasing HDL-C levels [46,47,48]. Recent research indicates heightened cholesterol absorption in young adults with T1DM [49], implying a potential efficacy of ezetimibe in this population, though this needs further investigation.

#### 3.2.2. Effects of Ezetimibe on Cardiovascular Outcomes

According to the IMPROVE-IT trial, more than 18,000 patients with established CAD (27% with DM) and elevated LDL-C levels were randomized to either statin monotherapy (simvastatin 40 mg/day) or statins plus ezetimibe (combination 10/40 mg/day) [50]. Patients on combined hypolipidemic medication (ezetimibe/simvastatin) showed greater decreases in LDL-C levels compared with simvastatin monotherapy (mean difference: −15.8 mg/dL; *p* < 0.001) and demonstrated modest but significant reductions in MACE incidence (32.7% vs. 34.7%; HR: 0.936; 95% CI: 0.89, 0.99; *p* = 0.016) [50]. Notably, these benefits were prominent in patients with DM [45,50]. The subgroup analysis of patients with DM showed an even greater decrease in the absolute risk of MACEs (HR: 0.86; 95% CI: 0.78, 0.94) after combined treatment (simvastatin plus ezetimibe) compared to the control group [50,51]. However, the combination therapy did not alter the mortality rate after acute coronary syndrome compared to statin monotherapy [50]. Indirect data from a large meta-analysis of 11 RCTs including over 109,000 high-CV-risk patients, among them patients with T2DM, revealed that the combination of statin and ezetimibe was effective in reducing MACEs compared to other combinations of statins with other lipid-lowering medications (niacin, CETP inhibitors, n-3 fatty acid, or fibrates). More precisely, a mean 8% reduction in MACEs was observed with the statin/ezetimibe combination arm when compared to the control group (RR: 0.92; 95% CI: 0.87, 0.97; *p* = 0.004) [52].

#### 3.2.3. Ezetimibe Side Effects

Ezetimibe is considered as a safe alternative hypolipidemic choice for diabetic patients with established CVD, as it has been associated with limited side effects either as a monotherapy or in combination with statins [53,54]. According to a meta-analysis pooling data from 18 trials and more than 14,500 patients, ezetimibe/statin combination did not elevate the risk of myalgias (risk difference −0.0033; 95% CI: −0.06, −0.01), rhabdomyolysis (risk difference −0.003; 95% CI: −0.01, 0.004), liver enzyme elevation (risk difference −0.003; 95% CI: −0.01, 0.005), or discontinuations due to serious side effects (risk difference −0.005; 95% CI: −0.03, 0.02) compared with statin monotherapy [55].

Overall, add-on therapy with ezetimibe for statin-treated patients with DM and established CAD aims to further reduce LDL-C levels below the ambitious target of 55 mg/dL, which is usually not achieved despite aggressive statin monotherapy [12]. Ezetimibe has been established as a first-line hypolipidemic medication in diabetic dyslipidemia not only for its modest but significant efficacy, but also for its safety, cost-effectiveness, and easy administration. Compared to novel PCSK9 inhibitors, ezetimibe clearly has a lower annual cost for an equivalent CV risk reduction in patients with DM and established CVD [56].

### 3.3. PCSK9 Inhibitors

Proprotein convertase subtilsin-kexin type 9 (PCSK9) inhibitors represent a relatively newer category of hypolipidemic treatment, comprising two entirely humanized monoclonal antibodies, evolocumab and alirocumab, which are designed to bind to the free-circulating PCSK9 protein [57,58]. This enzyme (hepatic protease) plays a pivotal role in facilitating the degradation of LDL-receptors (LDL-R) in the liver by internalizing them into hepatic lysosomes, promoting their destruction, and hindering their return to the hepatocyte membrane [58].

#### 3.3.1. Effects of PCSK9 Inhibitors on Lipid Profile

The improved hepatic clearance of LDL-C after PCSK9 inhibitor administration leads to a remarkable reduction (50–60%) in plasma LDL-C concentrations as an add-on therapy to statins [57]. PCSK9 inhibitors have been associated with significant CV benefits [59] and might be an effective alternative lipid-lowering medication among patients with CAD and DM [60]. Current guidelines strongly recommend PCSK9 inhibitors in individuals who cannot tolerate statins at all or those with very high CV risk and persistent LDL-C levels above the desired range despite a maximum-tolerated statin dosage in combination with ezetimibe [12]. Although previous trials have almost entirely focused on patients with familial hypercholesterolemia (FH) [61], diabetic patients were largely represented in those trials and sufficient data are available on their safety and efficacy profiles. In a recent meta-analysis pooling data from more than 10,000 patients, both alirocumab and evolocumab therapy achieved the LDL-C targets with great margins (87% and 98%, respectively) [61]. Another meta-analysis demonstrated that evolocumab significantly decreased plasma LDL-C levels in diabetic patients at a higher degree than the placebo (60%; 95% CI: 51, 69) and ezetimibe receivers (39%; 95% CI: 32, 47). Most importantly, evolocumab favorably altered the non-LDL lipid parameters of diabetic dyslipidemia (it increased HDL-C levels and decreased non-HDL-C, TC, and Lp(a) levels) [62]. In two studies (BANTING and BERSON), evolocumab administration in patients with T2DM was associated with significant reductions in atherogenic particles like TGs and non-HDL-C concentrations compared to controls [63,64]. Similar beneficial effects of alirocumab (as an add-on therapy to maximum-tolerated statin dosage) were observed on non-LDL lipid parameters of diabetic dyslipidemia compared with ezetimibe, fenofibrate, and non-hypolipidemic medication in the ODYSSEY DM-DYSLIPIDEMIA trial [65].

#### 3.3.2. Effects of PCSK9 Inhibitors on Cardiovascular Outcomes

Beyond the LDL-C target achievement, PCSK9 inhibitors (as an add-on therapy to statins, with or without ezetimibe) have demonstrated significant benefits in CV risk attenuation in diabetic patients with established CVD (including CAD), according to the findings of the two large landmark studies (FOURIER and ODYSSEY OUTCOMES) [66,67]. In the FOURIER trial, evolocumab significantly decreased MACEs compared to the placebo (HR: 0.83; 95% CI: 0.75, 0.93; *p* < 0.001) [66]. Similar findings were demonstrated among 5400 diabetic patients with established CAD in the ODYSSEY OUTCOMES trial with alirocumab [67]. These findings are of paramount importance regarding favorable effects on clinical endpoints in the very-high-risk group suffering from T2DM and CAD. Notably, both alirocumab and evolocumab have shown acceptable safety profiles with very limited adverse effects and a neutral impact on glycemic control in the diabetic population [68].

#### 3.3.3. Side Effects of PCSK9 Inhibitors

The administration of PCSK9 inhibitors seems to be safe, with the incidence of adverse effects uncommon [69]. Muscle toxicity and elevated liver enzymes, which are relatively common among statin use, have not been observed with the use of this drug category. The most common side effects include mild injection-site reactions [69]. Conflicting evidence exists as to whether treatment with PCSK9 inhibitors is associated with neurocognitive toxicity [66].

### 3.4. Fibrates

Fibrates demonstrate a favorable impact on diabetic dyslipidemia management through the activation of peroxisome proliferator-activated receptor α (PPAR-α).

#### 3.4.1. Effects of Fibrates on Lipid Profile

Fibrates collectively improve diabetic dyslipidemia since they decrease TG levels by 30–50%, total cholesterol by approximately 10%, and LDL-C levels by 10–30%, and increase HDL-C levels by 2–20% [8,10,70]. Before the widespread adoption of statins, fibrates were considered as a first-line hypolipidemic choice. There are in-class differences regarding their effects on triglyceride levels, as studies indicate that fenofibrate has a stronger effect in comparison to gemfibrozil [71,72]. The absence of class effects should be taken into consideration in clinical practice.

#### 3.4.2. Effects of Fibrates on Cardiovascular Outcomes

According to findings from two old large-scale trials (Veterans Affairs High-Density Lipoprotein Cholesterol Intervention Trial and the Helsinki Heart Study), gemfibrozil (a type of fibrate) demonstrated a noteworthy enhancement in primary and secondary CV prevention, both in the general and diabetic population [68,73]. Yet, in the period following the widespread use of statins, both the Action to Control Cardiovascular Risk in Diabetes study (ACCORD-Lipid) and the Fenofibrate Intervention and Event Lowering in Diabetes (FIELD) trial observed minimal additional benefits in CV outcomes after using fibrates (fenofibrate) routinely as an add-on treatment to statins for individuals with T2DM [74,75]. Focusing only on the diabetic population, with a pooled analysis of five trials among patients with significant elevated levels of TGs and decreased levels of HDL-C, fenofibrate was associated with reductions in CV risk (by approximately 35%) compared to the placebo [76]. In the recently published multicenter, double-blind, randomized controlled PROMINENT trial, enrolling 10,497 patients with T2DM (66.9% with established CVD), elevated TG levels (200–499 mg/dL), and low HDL-C levels (<40 mg/dL), pemafibrate improved lipidemic profiles (lower TGs and VLDL levels) but did not significantly reduce the incidence of CV events compared to the placebo [77]. In agreement, a previous meta-analysis pooling data from more than 45,000 patients treated with fibrates demonstrated a 10% reduction in the relative risk of major CV outcomes (*p* = 0.048) and a 13% reduction for CAD-related events (*p* < 0.001), with a neutral impact on mortality rates, either CV or total [78]. The beneficial effect of fibrates may be primarily driven by ameliorating TG and HDL-C levels. An old meta-analysis demonstrated the association of fibrates with the decreased risk of CV events in patients with either high TG or low HDL-C levels, or both (RR = 0.71, 95% CI: 0.62, 0.82; *p* < 0.001), but they showed a neutral effect on CV outcomes among patients without abnormal concentrations of TG and HDL-C (RR = 0.96; 95% CI: 0.85, 1.09; *p* = 0.53) [79].

Beyond the CV outcomes, it is noteworthy that fibrates (fenofibrate) have demonstrated a beneficial profile in the DM population as they can attenuate the progression of microvascular complications, particularly diabetic retinopathy. Findings from the FIELD trial showed that fenofibrate was associated with a decreased need for laser treatment for retinopathy compared with the placebo (*p* = 0.003) [74]. In addition, according to a subgroup analysis of the ACCORD-Lipid trial, the combined treatment of simvastatin plus fenofibrate was associated with a lower rate of progression of diabetic retinopathy compared with the control group (OR = 0.60; 95% CI: 0.42. 0.87; *p* = 0.006) [80]. Presumably, fibrates in combination with statin therapy management may have multiple clinical beneficial impacts on diabetic dyslipidemia, especially in patients with elevated TG levels and low HDL-C levels [81].

#### 3.4.3. Side Effects of Fibrates

Fibrate prescriptions have been associated with limited side effects, either as a monotherapy or in combination with statins. Myopathy and rhabdomyolysis are considered as primary clinical concerns, especially when fibrates are co-administered with statins [10]. Although these muscle-related complications are potentially life-threatening, their actual incidence is generally very low, especially for fenofibrate, in contrast to the combination of gemfibrozil with statins [82,83]. The risk of muscle-related side effects is heightened in individuals with kidney or liver diseases, advanced age, or those concurrently taking multiple medications [84]. Also, fibrates have been associated with a temporary and reversible elevation in serum creatinine levels [68,78,85]. In the ACCORD Lipid study, despite the observed rise in creatinine levels, fenofibrate treatment did not show a significant increase in the incidence of end-stage CKD needing dialysis. In contrast, compared to the control group, fenofibrate treatment was associated with a lower occurrence of microalbuminuria (38.2% vs. 41.6%; *p* = 0.01) and macroalbuminuria (10.5% vs. 12.3%; *p* = 0.04) [68]. Similarly, in the FIELD study, fenofibrate was associated with the attenuation of the progression rate of albuminuria [74]. In the PROMINENT trial, pemafribate was associated with more frequently reported renal adverse effects (HR = 1.12; 95% CI: 1.04, 1.20; *p* = 0.004) and with more frequently reported episodes of venous thromboembolism (HR = 2.05; 95% CI: 1.35, 3.17; *p* < 0.001) compared to the placebo [77]. The latter adverse effects remain to be proved.

### 3.5. Omega-3 Fatty Acids

The three most clinically relevant omega-3 polyunsaturated fatty acids (PUFAs) are (1) α-linolenic acid (ALA), (2) eicosapentaenoic acid (EPA), and (3) docosahexaenoic acid (DHA). The origins of oils containing omega-3 fatty acids are plant sources, fish, fish products, seeds, nuts, green leafy vegetables, and beans [86]. Ethyl eicosapentaenoic acid (E-EPA) is a purified form of eicosapentaenoic acid, an omega-3 polyunsaturated fatty acid derived from fish. Its triglyceride-lowering actions are proposed to be a result of decreased hepatic lipogenesis, an increased β-oxidation of fatty acids, the inhibition of enzymes involved in the triglyceride synthesis, and an increased expression of lipoprotein lipase [87].

#### 3.5.1. Effects of Omega-3 Fatty Acids on Lipid Profile

Administered omega-3 fatty acids are incorporated into cellular membranes and can modulate cellular signaling events, membrane protein function, and gene expression. They diminish the arachidonic acid-derived mediators of inflammation and the potential for platelets to produce thromboxane A2, a prothrombotic agent. They also reduce the secretion of cytokines involved in the amplification of the inflammatory pathways [88]. Regarding lipid parameters, the mechanisms of triglyceride lowering are still not fully elucidated. Presumably, they suppress lipogenic gene expression, increase the beta-oxidation of fatty acids, increase the expression of lipo-protein-lipase, and influence total body lipid accretion [87,89].

#### 3.5.2. Effects of Omega-3 Fatty Acids on Cardiovascular Outcomes

Omega-3 fatty acids may have a beneficial effect on CV outcomes, including ASCVD, through multiple underlying mechanisms such as triglyceride lowering and the attenuation of vascular inflammation [90,91]. According to a recent meta-analysis pooling data from 38 RCTs and more than 140.000 patients, omega-3 fatty acids significantly decreased CV outcomes (RR = 0.95, 95% CI: 0.92, 0.98; *p* = 0.002), including CV mortality (RR = 0.93; 95% CI: 0.88, 0.98; *p* = 0.01) [92]. Notably, EPA was associated with greater relative risk reductions compared to combination therapy with EPA plus DHA [86]. However, three recently published trials (STRENGTH, OMEMI, and ASCEND) have questioned the potential therapeutic efficacy of omega-3 fatty acids in preventing ASCVD [93,94,95]. The STRENGTH RCT included 13,078 patients with high CV risk (70% with DM). The administration of omega-3 fatty acids (EPA plus DHA) had a neutral impact on MACEs compared with corn oil (HR = 0.99; 95% CI: 0.90, 1.09; *p* = 0 .84) [92]. Similarly, in the findings from the OMEMI trial on 1014 patients with established CAD (20.7% with DM), 1.8 g n-3 PUFA (930 mg EPA and 660 mg DHA) administration did not decrease the incidence of primary outcomes (a composite of nonfatal AMI, unscheduled revascularization, stroke, all-cause death, and heart failure hospitalization after 2 years follow-up) compared to the placebo arm (receiving corn oil) (HR = 1.08; 95% CI: 0.82, 1.41; *p* = 0.60) [93]. In addition, the ASCEND trial included 15,480 diabetic patients without established CVD (primary prevention) and demonstrated that omega-3 fatty acid (1 g/daily) administration had a neutral effect on CV events (8.9% vs. 9.2%; RR = 0.97; 95% CI: 0.87, 1.08; *p* = 0.55) compared to the control group (olive oil) [95].

In the JELIS study (Japan EPA lipid-intervention study), more than 18,000 participants with cholesterol levels >250 mg/dL were randomized to receive statin therapy with or without 1.8 g E-EPA [96]. Each group consisted of 16% diabetic individuals. No significant differences were observed regarding the incidence of any major coronary event between the two treatment groups (HR: 0.86; 95% CI: 0.65, 1.15). No effect of diabetes presence was noticed.

Interestingly, a recent meta-analysis including three studies presented a pooled relative risk of 0.72 (95% CI: 0.62, 0.84) for nonfatal MI, indicating a protective effect of E-EPA [92]. Similarly, the relative risk of CAD was 0.73 (95% CI: 0.62, 0.85) after E-EPA therapy. However, no analysis was conducted for the diabetic sub-population, possibly because of the small number of participants. Studying the possible protective effects of E-EPA in larger studies with diabetic participants could provide a greater perspective on the role of this drug in the management of diabetic dyslipidemia.

#### 3.5.3. Omega-3 Fatty Acid Side Effects

Atrial fibrillation (AF) is considered a possible adverse effect of omega-3 fatty acid treatment [90]. Findings from the REDUCE-IT trail demonstrated an association of ethyl icosapentaenoic acid administration with AF incidence and hospitalization compared to the placebo arm (3.1% vs. 2.1%; *p* = 0.004) [90]. Similarly, in the STRENGTH trial, omega-3 fatty acid treatment significantly heightened the risk of new-onset AF compared to the control arm (2.2% vs.1.3%; HR = 1.69; 95% CI: 1.29, 2.21; *p* < 0.001) [94]. In addition, in the OMEMI trial, omega-3 fatty acid treatment was associated with a non-significant increase in the incidence of AF compared to the control group (7.2% vs. 4.0%; HR = 1.84; 95% CI: 0.98, 3.45; *p* = 0.06) [87]. Three recently published meta-analyses showed that omega-3 fatty acids are significantly associated with a dose-dependent increase in AF incidence rates [90,97,98]. On the other hand, according to findings from the VITAL-Rhythm trial, which evaluated the incidence of AF as a primary endpoint, omega-3 fatty acid administration did not increase AF episodes compared to the control group (3.7% vs. 3.4%; HR = 1.09; 95% CI: 0.96, 1.24; *p* = 0.19) [99]. Increased bleeding (major or minor) risk due to antiplatelet action has not been confirmed.

The impacts of classic hypolipidemic agents on lipid parameters, CV outcomes, and side effects are summarized in Table 1.

## 4. Novel Lipid-Lowering Medications

Novel hypolipidemic medications have emerged with potential cardiovascular benefits for diabetic patients (Table 2).

### 4.1. Bempedoic Acid

Bempedoic acid is an oral, relatively novel compound that exerts hypolipidemic effects through the inhibition of adenosine triphosphate citrate lyase action. In the pathway of cholesterol synthesis, this enzyme is located upstream of HMG-CoA reductase [100].

#### 4.1.1. Effects of Bempedoic Acid on Lipid Profile

Recent data from clinical trials have investigated the effects of bempedoic acid on lipid profiles. A pooled analysis of four RCTs—CLEAR Harmony, CLEAR Wisdom, CLEAR Tranquility, and CLEAR Serenity—aimed to assess the efficacy of bempedoic acid (180 mg per day) versus placebo to reduce LDL-C levels in patients with hypercholesterolemia and ASCVD (97% of the study population) or heterogenous FH (HeFH) (3%) [101]. Among them, the diabetic patients (N = 839) randomly assigned to receive bempedoic acid experienced a mean reduction of 19% (95% CI: −22.2, −15.8) in LDL-C levels after 12 weeks of treatment, compared to the placebo group. Interestingly, the mean reduction in LDL-C levels for the non-diabetic sub-population was significantly lower than that observed in the diabetic one. In the same study, a second sub-group analysis focused on patients with statin intolerance. Among them, diabetic participants receiving bempedoic acid (N = 96) exhibited lower LDL-C levels by 18% (95% CI: −24.3, −11.8) than the non-diabetic group (N = 40). Based on the glycemic status of each participant (diabetes, prediabetes, normoglycemia), another pooled analysis of the same studies was conducted, presenting a similar degree of LDL-C decrease after treatment with bempedoic acid for the diabetic sub-population with concomitant ASCVD/HeFH and the diabetic statin-intolerant subgroup (−19.1; *p* < 0.001 for both groups) [102].

A post hoc analysis of these studies identified that diabetic patients may be more likely to benefit from bempedoic acid in terms of LDL-C reduction, the amount of which may reach 36% in bempedoic acid-treated patients (OR: 1.35; 95% CI: 1.11, 1.64; *p* < 0.001) [103]. Another RCT aimed to investigate the additive effect of bempedoic acid plus ezetimibe in the reduction of LDL-C levels, enrolling individuals at high CV risk (ASCVD, HeFH, or multiple CV risk factors) [104]. The combined treatment for the small diabetic arm (35 diabetic individuals) significantly reduced their LDL-C levels (~30%) in comparison to the placebo group (*p* < 0.001).

#### 4.1.2. Effects of Bempedoic Acid on Cardiovascular Outcomes

From the perspective of clinical endpoints, recent data support the protective role of bempedoic acid in the primary and secondary prevention of ASCVD. In the CLEAR Outcomes study, the administration of bempedoic acid was associated with a statistically significant 23% reduction in fatal or nonfatal MI (HR: 0.77; 95% CI: 0.66, 0.91; *p* = 0.002) and a 19% lower risk of coronary revascularization (HR: 0.81; 95% CI: 0.72, 0.92; *p* = 0.001) in statin-intolerant patients versus controls [105]. However, these constitute secondary endpoints of the study and should be interpreted with caution. The same study group compared the efficacy of bempedoic acid versus placebo in 4206 statin-intolerant individuals with LDL-C > 100 mg/dL at high risk for a first CV event [106]. While similarly to above administration of bempedoic acid seems to be associated with a significant mean reduction in risk of fatal or nonfatal MI (HR: 0.61; 95% CI: 0.39, 0.98), a statistically non-significant reduction was observed regarding the risk of coronary revascularization (HR: 0.71; 95% CI: 0.49, 1.03). Using the Cholesterol Treatment Trialists’ coefficient and the baseline SMART model, a post hoc analysis of the four CLEAR trials tried to identify the impact on 10-year CV event risk [104]. Participants who were on maximally tolerated statin doses and those identified as statin-intolerant were divided into two cohorts. Bempedoic acid treatment seemed to confer a mean 3.3% reduction in 10-year CV risk for the maximum-statin-dose group (95% CI: −3.7, −2.9; *p* < 0.001) and a 6% reduction for the statin-intolerant group (95% CI: −7.7, −4.3; *p* < 0.001) compared to placebo.

It is evident that bempedoic acid is effective in the reduction of LDL-C levels in the diabetic population with potential CV benefits, especially among patients with ASCVD. However, more studies need to be conducted to evaluate its effect in patients with CAD and diabetic dyslipidemia.

#### 4.1.3. Side Effects of Bempedoic Acid

The overall use of bempedoic acid appears to be safe with mild or moderate adverse effects, including gout and hyperuricemia, cholelithiasis, an increase in aminotransferase levels, and renal impairment [104,105]. An increased incidence of muscle pain and headaches is questionable. However, it is important to note that studies investigating the safety of bempedoic acid have a relatively short follow-up period and its long-term effects are still unknown.

### 4.2. Inclisiran

Inclisiran is a small interfering RNA (siRNA) molecule that prevents the expression of the PCSK9 gene [107]. Due to its conjugation with triantennary N-acetylgalactosamine carbohydrates, it has a high specificity to hepatocytes, which allows its specific action.

#### 4.2.1. Effects of Inclisiran on Lipid Profile

So far, the ORION-10 and ORION-11 trials, two phase III RCTs conducted in the United States, Europe, and South Africa, have investigated the possible effects of inclisiran on dyslipidemia [108]. Each of these two studies recruited about 1600 participants. The inclusion criterion for the first study was LDL-C > 70 mg/dL, while for the second it was ASCVD or T2DM or familial hypercholesterolemia or 10-year CV risk above 20%. Both studies included a diabetic sub-population (45% in ORION-10 and 35% in ORION-11) who were randomized to receive 284 mg inclisiran subcutaneous or the placebo. The injections were carried out on day 1, 30, 270, and 450, and participants were followed-up for another 90 days. Regarding the diabetic sub-population in these two studies, inclisiran administration was associated with a mean reduction in LDL-C levels of 55.2% and 56.3% in comparison to the placebo group (95% CI: −60.6, −49.9; 95% CI: −61.1, −51.5, respectively). Similar percentages of LDL-C reduction were present in ORION-1, a phase II RCT including 67 diabetic individuals in which inclisiran was administered at different doses (100, 200, 300 mg) at intervals of 90 days [109]. Additionally, a statistically significant amelioration in HDL-C levels as well as apolipoprotein B and lipoprotein (a) was observed [110].

#### 4.2.2. Effects of Inclisiran on Cardiovascular Outcomes

A pooled analysis of the ORION-9, ORION-10, and ORION-11 studies, including more than 3500 individuals with high CV risk and elevated LDL-C levels, reported after inclisiran treatment a statistically non-significant reduction in the incidence of MI, fatal or nonfatal (HR: 0.81; 95% CI: 0.51, 1.29; *p* = 0.38) [111]. In the same study, inclisiran therapy significantly reduced the risk of MACEs, including nonfatal MI (HR: 0.75; 95% CI: 0.60, 0.94; *p* = 0.01). Due to these controversial results and the absence of a sub-group analysis in the T2DM population, the results of the ongoing phase III RCTs are eagerly awaited, especially among diabetic individuals.

#### 4.2.3. Side Effects of Inclisiran

Inclisiran administration has been associated with mild and self-limited adverse effects [112]. Local injection-site reactions are mild or moderate in intensity and constitute the most common side effects [108].

### 4.3. Ethyl Icosapentaenoic Acid

**Figure 2:**
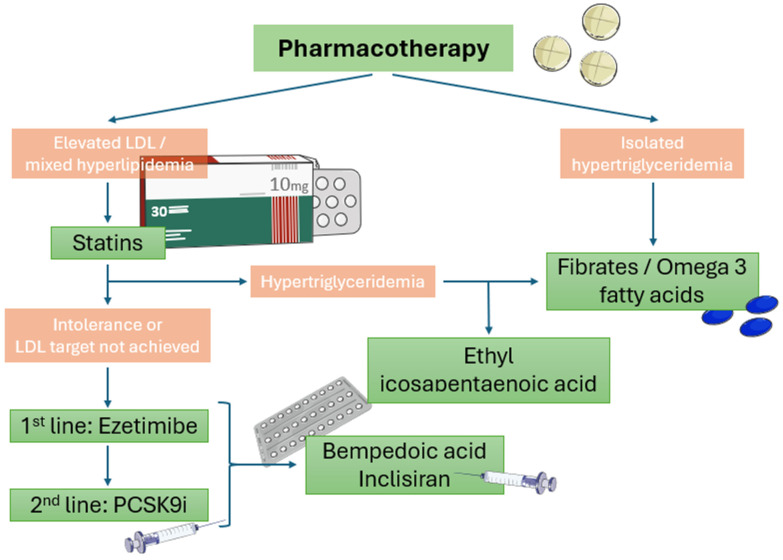
Algorithm of pharmaceutical lipid-lowering therapy in diabetic patients with established CAD or high risk of CAD.

According to 2023 ESC guidelines for the management of ASCVDs in patients with diabetes, omega-3 fatty acids are suggested for hypertriglyceridemia management as an add-on therapy to statins [13]. More specifically, ethyl eicosapentaenoic acid, a highly purified stable ester of EPA, is preferable (2 g/bid) for triglyceride level lowering [13] due to its beneficial impact on CV outcomes (including CV mortality) compared to placebo (HR = 0.75; 95% CI: 0.68, 0.83; *p* < 0.001), according to the recently published REDUCE-IT trial which had a follow-up period of 4.9 years [113]. Diabetic individuals receiving E-EPA had a 23% decreased risk of primary composite endpoints, namely CV death, nonfatal MI, nonfatal stroke, coronary revascularization, or unstable angina (HR: 0.77; 95% CI: 0.68, 0.87). Given that the control group received mineral oil which was associated with a mild elevation in LDL-C and CRP levels, the favorable effects of ethyl eicosapentaenoic acid remained significant [114,115], indicating a promising alternative hypolipidemic treatment option for diabetic dyslipidemia. Its use has been associated with increased incidences of atrial fibrillation, peripheral edema, and constipation, although these adverse effects are rare. The most common side effects are mild (Figure 2).

## 5. Antilipidemic Effects of Anti-Diabetic and Anti-Hypertensive Medications

Sodium-glucose cotransporter 2 inhibitors (SGLT2is), or gliflozins, are a group of anti-diabetic drugs that, apart from glycemic control, seem to exert a neutral effect on lipid levels through a number of proposed but not yet fully understood mechanisms [116]. A recent meta-analysis of 48 trials aimed to investigate the possible lipid-lowering effects of SGLT2i, presenting a pooled increase in LDL-C levels by 3.9 mg/dL, as well as in HDL-C levels, and a decrease in triglyceride levels, but with high heterogeneity between studies [117]. Similar results are presented by other meta-analyses [118,119]. A pooled analysis of 27 studies with diabetic patients at increased risk of CAD revealed a 23% lower risk for MI (HR: 0.77; 95% CI: 0.60, 0.99), but this was not a class effect, since among the tested SGLT2is (canagliflozin, dapagliflozin, and empagliflozin), only canagliflozin showed an inverse association with MI incidence [119]. Glucagon-like peptide-1 (GLP-1) receptor antagonists (RAs) constitute a group of injectable medications that, beyond their glycemic control, have shown controversial changes in lipidemic markers and protect against ASCVD [116]. A meta-analysis of 57 studies investigated the pooled effect of GLP-1 RA on cholesterol levels, indicating a statistically significant beneficial effect only on LDL-C levels, which was non-significant when sub-group analysis was performed in diabetic individuals [118]. Regarding the possible effects of GLP-1 RA administration on nonfatal MI, this meta-analysis failed to identify a statistically significant association (HR: 0.91; 95% CI: 0.81, 1.01; *p* = 0.008); however, a reduction in the risk of MACEs was identified (HR: 0.86; 95% CI: 0.79, 0.94; *p* = 0.006) [120]. Dipeptidyl peptidase-4 inhibitors (DPP4i) are a class of glucose-lowering drugs with possible benefits on lipid blood levels [116]. A recent meta-analysis did not identify an association between the use of DPP4i and cholesterol levels [118], while a study which reported results on diabetic participants identified a significant reduction in HDL-C levels (mean difference: −6.00; 95% CI: −8.43, −3.57) [121]. In conclusion, it seems that SGLT2is exhibit a mild hypolipidemic action. Regarding GLP-1 RA, current evidence suggests they do not significantly influence lipid profiles and their role in CAD needs to be investigated in greater depth. Existing clinical studies have not identified an association between DPP4i and lipid levels, with a possible exception noted for HDL-C levels.

The effects of anti-hypertensive medication on lipid levels have been investigated but vary between each drug category. A dose-dependent hazard effect of thiazide diuretics has been observed on LDL-C levels and to a lower extent on triglyceride levels, with high doses inducing an increase of 5 to 10% in LDL-C levels [122,123]. Beta blockers exert different effects on lipid profiles depending on each drug [122,124]. Some of them have neutral effects (e.g., nebivolol) [125], while others are associated with an increase in triglyceride levels (e.g., metoprolol and propranolol) [124]. A neutral or modest beneficial association is observed for the anti-hypertensive drug classes of angiotensin-converting enzyme inhibitors (ACEIs), angiotensin receptor blockers (ARB), calcium channel blockers, and alpha blockers [122,126]. Interestingly, the use of ACEIs when combined with diuretics seems to minimize or prevent the negative effects on lipid profiles induced by the latter drug category [127]. Regarding ARBs, a possible favorable effect of telmisartan administration on lipid parameters, such as total cholesterol, LDL-C, and triglyceride levels, was observed when compared with eprosartan, an agent belonging to the same drug category [128]. In general, anti-hypertensive medication seems to not exert any negative influence on lipid metabolism, with the possible exceptions of thiazide diuretics and some beta blockers.

## 6. Future Prospectives

At present, there is rapidly growing evidence of multiple pharmaceutical agents with remarkable hypolipidemic effects with potential cardiovascular protection. Diabetic dyslipidemia is a demanding disorder which requires prompt and effective therapy. Statins, and to a lesser extent ezetimibe, remain the first-line therapy in diabetic patients with high CV risk or established CVD. Other pharmaceutical agents, like PCSK9 inhibitors, have shown resounding hypolipidemic effects, but their CV impact needs further validation. In the near future, the threshold for prescribing these drugs to diabetic patients will lower and a wider spectrum of patients will receive them. In the context of precision medicine, we believe that individualized hypolipidemic therapy will be the common approach in the diabetic population. Finally, we are looking for new medications targeting HDL elevation. Previous research attempts did not confer applicable results, and medications enhancing HDL profiles are still the missing piece of the diabetic dyslipidemia puzzle.

## 7. Conclusions

In conclusion, ongoing research is exploring the efficacy of new drugs for dyslipidemia and their protective effects against CAD. Statins and the complementary administration of ezetimibe play a predominant cardioprotective role in diabetic patients with CAD or at risk of CAD. PCKS9 inhibitors with their strong lipid-lowering impact may reduce MACEs in this population. Fibrates or omega-3 fatty acids may exert modest effects on CV outcomes, despite beneficial alterations in lipid profiles. On the other hand, further investigation is warranted to elucidate the promising protective effects of E-EPA. Bempedoic acid and inclisiran, both approved by the US Food and Drug Association (FDA) and the European Medicines Agency (EMA), have a promising role in this direction. Despite the existing body of evidence, their examination within expansive diabetic cohorts is limited [129]. As a result, their potential therapeutic role in the management of diabetic dyslipidemia is still not adequately documented. Given the heightened CV risk among individuals with T2DM, more decisive results would be of great importance in the utility of these drugs.

## Figures and Tables

**Table 1:** Beneficial cardiovascular outcomes and the most common side effects of classical hypolipidemic therapies in diabetic patients.

Drugs	Mechanisms	Lipid Changes and Clinical Outcomes	Side Effects
**Statins**	Inhibition of HMG-CoA reductase, ↓↓↓ endogenous production of LDL-C, ↑ clearance of atherogenic lipoproteins	↓↓↓ LDL-C, ↓↓ sdLDL, ↔ HDL-C, ↓ TG ↓↓ CV morbidity and mortality in diabetic patients, especially those with already-existing CAD Dose-dependent manner of LDL-C reduction Greater clinical effect by high-intensity statins	Fatigue, myalgias, nervous system symptoms Less frequent: significant elevations in CPK/rhabdomyolysis
**Ezetimibe**	Suppression of NPCL1 protein	↓↓ LDL-C, ↓ TG, ↓ sdLDL, ↑ HDL-C ↓↓ MACE ↔ mortality after ACS	-
**PCSK9 inhibitors**	Binding of PCSK9 facilitates LDL receptor degradation in liver	↓↓↓ LDL-C, ↑ HDL-C, ↓ TG, ↓ non-HDL-C, ↓ Lp(a) ↓↓ CV morbidity	Limited adverse effects
**Fibrates**	Activation of PPAR-α	↓↓ TG, ↓ total cholesterol, ↑ HDL-C ↓↓ or ↔ CV events	Myopathy, rhabdomyolysis ↑ CPK, renal impairment, VTE (pemafribate)
**Omega-3**	↓↓ hepatic lipogenesis, ↑ β-oxidation of fatty acids, ↓ enzymes mediating TG synthesis, ↑ lipoprotein lipase	↓↓ TGs ↓ or ↔ CV morbidity and mortality	Atrial fibrillation

ACS, acute coronary syndrome; CAD, coronary artery disease; CPK, creatine phosphokinase; CV, cardiovascular; HDL-C, high-density lipoprotein cholesterol; HMG-CoA, 3-hydroxy-3-methyl-glutaryl-coenzyme A; LDL-C, low-density lipoprotein cholesterol; LP(a), lipoprotein (a); MACE, major adverse cardiovascular event; NPCL1, Niemann-Pick C1-like 1; PCSK9, proprotein convertase subtilsin-kexin type 9; PPAR-α, peroxisome proliferator-activated receptor α; sdLDL, small dense low-density lipoprotein; TG, triglycerides; VTE: venous thromboembolism. ↓↓↓ remarkable decrease, ↓↓ significant decrease, ↓ slight non-significant decrease, ↑ slight non-significant increase, ↔ unaltered.

**Table 2:** Novel hypolipidemic therapies with indications of cardiovascular benefits for diabetic patients.

Drug	Mechanisms	Lipid Profile Changes and Clinical Outcomes	Adverse Effects
**Bempedoic acid** **Dose:** 180 mg O.D.	Inhibition of ATP citrate lyase action, located upstream of the HMGCR	↓↓ LDL-C ↓↓ risk of fatal or nonfatal MI ↓ or ↔ risk of coronary revascularization ↓↓ 10-year cardiovascular risk	Mild adverse effects: gout, hyperuricemia, cholelithiasis, ↑ aminotransferase, kidney impairment, ± headaches, myalgias
**Inclisiran** **Dose:** 284 mg SC injection, repeat in 3 mo and then every 6 mo	↓↓↓ expression of PCSK9 gene High specificity to hepatocytes	↓↓↓ LDL-C ↑ HDL-C, ↓ Lp(a), ApoB ↔ risk of fatal or nonfatal MI ↓↓ risk of MACE	No serious adverse effects, similar to placebo group
**Ethyl eicosapentaenoic acid** **Dose:** 2 g B.D.	↓↓ hepatic lipogenesis ↑↑ β-oxidation of fatty acids ↓↓ TG synthesis ↑↑ expression of lipoprotein lipase	↓↓ risk of composite endpoint: CV death, nonfatal MI, nonfatal stroke, coronary revascularization or unstable angina ↔ risk of major coronary event ↓↓ risk of nonfatal MI, CAD	Mild adverse effects: atrial fibrillation, peripheral edema, constipation

ApoB, apolipoprotein B; ATP, adenosine triphosphate; B.D., twice daily; CAD, coronary artery disease; CV, cardiovascular; HDL-C: high-density lipoprotein cholesterol; HMGCR, 3-hydroxy-3-methyl-glutaryl-coenzyme A reductase; LDL-C, low-density lipoprotein cholesterol; Lp(a), lipoprotein (a); MACE, major adverse cardiovascular event; MI, myocardial infarction; mo, months; O.D., once daily; PCSK9, proprotein convertase subtilsin-kexin type 9; SC, subcutaneous; TG, triglycerides. ↓↓↓ remarkable decrease, ↓↓ significant decrease, ↓ slight non-significant decrease, ↑↑ significant increase, ↑ slight non-significant increase, ↔ unaltered
